# Evaluating Horse Owner Expertise and Professional Use of Auxiliary Reins during Horse Riding

**DOI:** 10.3390/ani11072146

**Published:** 2021-07-20

**Authors:** Heidrun Gehlen, Julia Puhlmann, Roswitha Merle, Christa Thöne-Reineke

**Affiliations:** 1 Equine Clinic, Veterinary Department, Freie Universitaet Berlin, 14163 Berlin, Germany; Julia.Puhlmann@fu-berlin.de; 2 Veterinary Department, Institute for Veterinary Epidemiology and Biostatistics, Freie Universitaet Berlin, 14163 Berlin, Germany; Roswitha.Merle@fu-berlin.de; 3 Animal Behavior and Laboratory Animal Science, Veterinary Department, Institute of Animal Welfare, Freie Universitaet Berlin, 14163 Berlin, Germany; Christa.Thoene-Reineke@fu-berlin.de

**Keywords:** horse, training, auxiliary reins, draw reins, sliding ring martingale

## Abstract

**Simple Summary:**

Auxiliary reins, which function as mechanical training aids that exert influence on the posture of the horse, are often criticized, especially if they are used incorrectly and against animal welfare. The aim of this paper was to investigate, with an online questionnaire, how much knowledge horse owners have regarding auxiliary reins and whether they use them appropriately. In our study, the running side rein was the most popular auxiliary rein when working from the ground and the sliding ring martingale was the most popular for equestrian activities. Half of the participants did not change the auxiliary rein during the entire training session and most participants adjusted their horse too tightly and did not change anything at that time despite the related breathing problems. The study showed that most participants used the reins responsibly, but there is still a need for clarification of their correct application regarding animal welfare and training physiology among horse owners.

**Abstract:**

Auxiliary reins are commonly used for the training of riders and horses as well as in daily training. They are often criticized when used incorrectly, as they will not help and can harm the horse by causing overwork, accidents, and injuries, which harm the horse in the long term. They also often conceal causal rider problems while trying to achieve quick success. The aim of this paper was to investigate, with an online horse-owner questionnaire, which and how often auxiliary reins were used and whether they were used appropriately. Only participants who were currently using auxiliary reins were selected. Consequently, 823 participants were questioned, of which 362 were currently using auxiliary reins at least every two weeks. Auxiliary reins were mainly used according to their discipline: the running side rein was the most popular when working from the ground and the sliding ring martingale was the most popular for ridden equestrian activities. Most of the test subjects only attached the auxiliary reins after the warm-up phase, but half of the participants did not change them during the entire training session. Most participants (75%) could at least identify what the correct head position of the horse should look like. However, there were still too many (50%) who adjusted their horse too tightly and did not change anything at that time despite the related breathing problems. The study found that most participants used the reins responsibly, but there is still a need for clarification and information relating to the functions of the different auxiliary reins among horse owners.

## 1. Introduction

**Figure 1:**
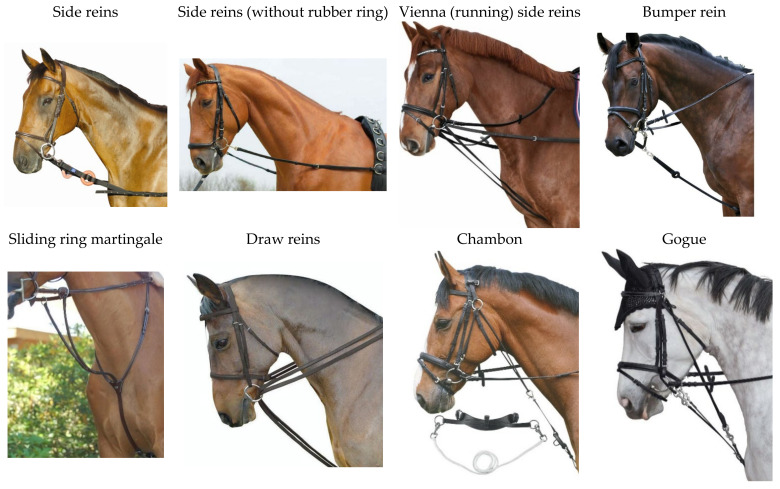
Different auxiliary reins used for equids as training aids.

Auxiliary reins, such as running side reins, lunging reins, side reins, draw reins, Chambon, Gogue, and the sliding ring martingale (Figure 1), are mechanical equine training aids which are used for various reasons. The most common auxiliary reins used for equids are running side reins (also known as *Wiener Zügel*: Viennese reins) and draw reins (Figure 1).

*Running side* and *lunging reins* slide through the snaffle rings and are attached to the lunging/saddle girth and saddle; therefore, they are designed to help the horse into a forward-downward extension. The reins restrict and restrain the position and movement of the horse’s head and neck, and influence the movement of the back and stride characteristics. Rhodin et al. demonstrated that when the horse’s head and neck were restrained in a high position, the stride length and flexion and extension of the caudal back were significantly reduced [1]. The use of *side reins* is also anecdotally advocated to promote the development of the horse’s dorsal muscles or ‘top line’ by encouraging the horse to work in a more collected frame, increasing engagement of the abdominal musculature and the hind limbs. However, evaluation of muscle activity within the horse’s spinal muscle, longissimus dorsi, found the use of side reins did not increase the muscle workload [2]. *Side reins*, similar to the running side and lunging reins, are unsuitable for use in cross-country and should not be used for jumping because they are firmly attached to the snaffle ring [3,4,5].

The *draw rein* is buckled to the side or ventral of the girth, similar to running side or lunging reins, and runs over the snaffle rings into the rider’s hand. *Draw reins* act as levers and increase the effect of the rider’s hand [1]; they are used to increase collection by shifting the horse’s centre of gravity caudally, thereby increasing weight bearing on the hind limbs and developing increasing suppleness in the head and neck by facilitating lateral flexion [6]. Studies have shown that the combined use of draw reins with normal reins can facilitate reduced head and neck angles to generate the desired effect of increased collection characterized by higher impulses in the hind limb and increased flexion of the hock and extension of the hip joint [6,7]. Therefore, draw reins should only be used in conjunction with the horse’s normal rein incorporating pressure-release and not persistent contact to enhance performance.

The *Chambon* and *Gogue*, on the other hand, are classically used for lunging. They run between the front legs to a neck piece and work with pressure on the neck or flews (Gogue). The pressure on the neck is to prevent the head from being carried too high [8,9]. This can lead to recalcitrance in some horses [8]. The gogue is buckled almost like the chambon. The only difference is that the two lower carabiners are not buckled into the bit rings, but passed through and, like the upper carabiners, are hooked onto the belt ring that comes from the lunging belt.

Only the *sliding ring* *martingale* is suitable for cross-country riding and jumping. It does not exert direct pressure on the horse’s bit but prevents the horse from raising its head too high by directing the reins downwards. A ring through which the rein is passed achieves this. The rings are each attached to the girth by a strap that joins in the direction of the girth between the horse’s legs. To keep them in place, they are passed through a neck strap that comes to rest in front of the shoulder. The rings themselves are kept from snagging on the bridle rings by sliders [9].

Many equestrians are proficient at using training aids. However, regardless of the qualification level, unintentional neglect due to owner or rider ignorance [10] and inflated confidence in their equine-related knowledge (Dunning-Kruger effect) [11] could result in people utilising equine training aids inappropriately and unintentionally comprising their horse’s welfare and performance [12]. Against this background, this study aimed to investigate to what extent horse owners can use auxiliary reins correctly, and whether they have expert knowledge about the correct buckling method and the way it works. It is also important to find out whether there are differences between amateur and professional riders in this aspect. It was asked how long auxiliary reins had already been used in training and whether there was a willingness to use them only for a short period of time.

## 2. Materials and Methods

A quantitative method from empirical social research was mainly used to generate the data for the present study. An online questionnaire was developed using a predefined answer grid and the results were mainly analyzed descriptively and, to a lesser extent, statistically. One question from the questionnaire was open-ended: “28. What do you want to achieve with the use of auxiliary reins?”. Written answers were categorized into nine groups including the category “others”. This allowed the respondents to answer freely and without bias on this point. All other questions were closed single or multiple choice questions and some were of quantitative nature. The total of 49 questions included (i) information on the horse, (ii) training data, (iii) auxiliary reins, and (iv) demographic data.

The questionnaire was distributed via social media and e-mails to 24 horse-specific groups from Germany and Austria. With the help of a specially created QR code, the link could be made accessible for response in eleven randomly selected riding stables with German Equestrian Federation (FN) identification. Participation could not be traced back since it was exclusively anonymous in accordance with data protection requirements. Horse owners, trainers, and riding partners who used auxiliary reins when riding were surveyed.

The survey was created with the open source software “LimeSurvey” of the Free University of Berlin. The questionnaire could be adapted very specifically to the target group. The data collected were stored on the servers of the Free University of Berlin. No personal data were stored in order to guarantee anonymity during the survey. The questionnaire was discussed with experts before publication and revised at the appropriate points to enable an optimal development.

The duration of the online survey was five weeks and covered the period from early November to mid-December 2019.

The evaluation and graphic representation of the online questionnaire was conducted with the help of the statistics program ‘SPSS 26′ on the Macintosh operating system. Figures were created with Microsoft Excel. Frequencies, mean values, and standard deviations were given in SPSS. Finally, associations between qualitative variables were tested for significance with Pearson’s chi-square test and Fisher’s exact test (if more than 25% of cells had expected values below 5). These tests were used to investigate if the objective of using auxiliary reins differed between professional and recreational riders (chi-square test), if the duration of the use of auxiliary reins differed between different intentions (Fisher’s exact test), and if the use of auxiliary reins depended on lessons with a trainer or not (chi-squared test). The significance level was set at 5%.

Respondents who stated that they did not use auxiliary reins regularly were excluded from answering questions about frequency of use per week and frequency of reasons for use.

## 3. Results

**Figure 2:**
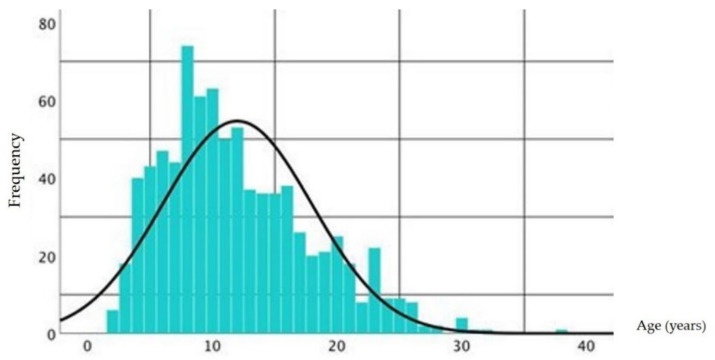
Age distribution of the horses in years. The frequency of the horses’ age is shown with a normal distribution curve superimposed.

A total of 1026 people took part in the survey. Of these, 823 complete questionnaires (80.21%) were used for the study. First of all, general information about the horse was provided. The questionnaire referred to only one horse per participant. The horses were between 2 and 38 years old, an average of 11.98 years old (Figure 2). A total of 56.6% (*n* = 466) of the participants had geldings, 41.1% (*n* = 338) mares, and 2.3% (*n* = 19) stallions.

Most of the horses were warmbloods (58.7%, *n* = 483) and ponies (17.4%, *n* = 143). Other breeds were represented by less than 10% (*n* = 94). The vast majority of horses (71.6%, *n* = 762) were already trained.

Most horses were ridden (89.3%, *n* = 735), while 8% (*n* = 66) were used for ground work and less than 2% were either driven (*n* = 13) or used as vaulters (*n* = 9). A total of 41.7% (*n* = 343) of the participants took part in competitions, mostly competing in minor-level performance classes.

The survey then aimed to determine the training behaviour of the participants. Most of the survey participants stated that they trained with their horse three to four days (44%, *n* = 362) or five to six days (41.9%, *n* = 345) a week. Only 3.8% (*n* = 31) worked with their horse every day and 10.3% (*n* = 85) one to two days a week. The bridles most commonly used were the English and the English-combined halters (29.8%, *n* = 245 and 29.3%, *n* = 241, respectively). The loose ring snaffle (single broken 24.7%, *n* = 203 and double broken 27.6%, *n* = 227) was used most frequently. The double broken olive head bit was also used by 14.3% (*n* = 118) of the respondents. All other bits were used by less than 10% each.

**Figure 3:**
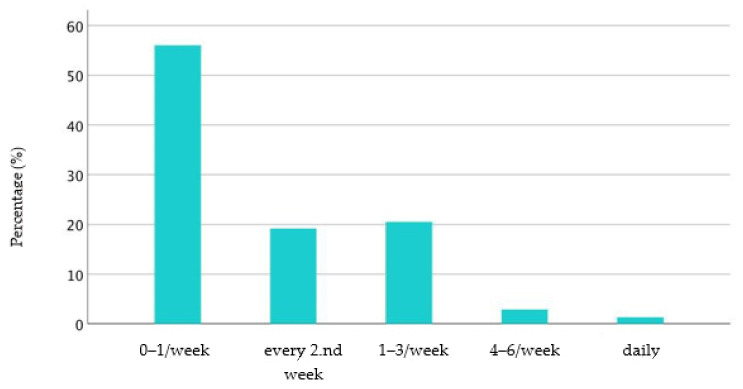
Frequency of use of auxiliary reins per week in %.

When asked about the use of auxiliary reins, 56% (*n* = 461) of respondents said they did not use them regularly. Respondents who stated this were excluded from answering further questions. Furthermore, 19.2% (*n* = 158) of the respondents claimed to use auxiliary reins only once every fortnight, whereas 20.5% (*n* = 169) used them one to three times a week. By contrast, eleven respondents (1.3%) used auxiliary reins daily (Figure 3).

This was followed by the question of how important the use of auxiliary reins in training was for the respondents: 40.2% (*n* = 145) considered their use important, and 36.3% (*n* = 131) were neutral towards them. The participants mainly used auxiliary reins for lunging (81.8%, *n* = 296 or 49.2% with multiple answers), but also for riding (28.2%, *n* = 102), show jumping (22.1%, *n* = 80), and cross-country (18.8%, *n* = 68).

Subsequently, participants were asked about the use of auxiliary reins. The first step was to find out which auxiliary reins were used. The running side reins were used most frequently by the respondents (34.3%, *n* = 55), while 29.6% (*n* = 62) used a sliding ring martingale. The two classic auxiliary reins, the side reins and the lunging reins, were only used by 10% (*n* = 16) of the respondents. Newer auxiliary reins, such as Chambon and Gogue, were used by 4.6% (*n* = 4) of the respondents and draw reins by 5.9% (*n* = 20).

Running side reins were used for lunging in 68.9% (*n* = 206) of the cases. This was followed by 22.6% (*n* = 67) who used side and 21.6% (*n* = 64) who used lunging reins (multiple answers possible). The other auxiliary reins were used by less than 10% of the respondents.

Far fewer auxiliary reins were used for riding (*n* = 166). Here, too, the running side rein was often used (53.9%); only the sliding ring martingale was used more frequently (60.8%). The draw reins were used third most often (19.6%), while all other auxiliary reins were used less than 10% of the time.

Auxiliary reins were used in 85 cases during jumping. Here, the sliding ring martingale was mainly used (93.8%, *n* = 75), but never Chambon nor Gogue. Lunging reins, running side reins, and draw reins were only used in individual cases. The data for cross-country riding were similar. Auxiliary reins were used by 76 riders: the sliding ring martingale was used most often (92.6%, *n* = 63), as well as the draw reins (4.4%, *n* = 3) and others (with the exception of Chambon and Gogue).

Regarding eventing, however, hardly any auxiliary reins were used (*n* = 30). The sliding ring martingale was also used most frequently (85.2%, *n* = 23), with the rest being made up of draw, side, and running side reins.

By contrast, only two participants used auxiliary reins in trekking and endurance competitions: side, running side, and draw reins, and sliding ring martingales were used alternately. Auxiliary reins were used a total of 21 times in other disciplines. Running side reins were predominantly used in 40% of the cases and sliding ring martingales in 33.3%. These were followed by side and lunging reins, each in 20%, and draw reins in individual cases. If the respondents indicated that they used auxiliary reins when riding, they were asked where they were used. Most respondents (86.3%, *n* = 88) said that they used auxiliary reins when riding in the arena, while the rest (13.7%, *n =* 14) used them when riding cross-country.

In addition, they were asked for information regarding why auxiliary reins were used. A total of 52% (*n* = 93) of the riders used auxiliary reins for self-correction and support and 48% (*n* = 86) for correcting the horse.

This was followed by the question of when the auxiliary reins were buckled in. Most of the participants did this after the warm-up phase (71.1%, *n* = 253), while some had already buckled them in the stable aisle (9.3%, *n* = 33), and the rest before the start of the training session (14.3%). The aim was also to find out whether the respondents change the auxiliary reins during a training session. Half of the participants (*n* = 181) answered this in the affirmative, and the remaining half left them unchanged the whole time. Those who made a change did so in 48.1% (*n* = 87) of the cases after the warm-up phase or after changing sides (43.6%).

The auxiliary reins were changed after the training session or during changes of pace in only about 20% of cases. Three test respondents stated that they specifically changed something during the training. When the participants were asked what they used auxiliary reins for, 30.4% (*n* = 110) said they wanted to improve forward-downward extension, ensuring contact was the reason for 24.9% (*n* = 90), and, finally, a significant number of participants also wanted to ensure support for novice riders, take away uncertainty in the cross-country, or fulfil an instruction obligation (18.5%); 17.7% (*n* = 64) hoped to improve the arching of the back, to directly control the pace (11.3%, *n* = 41), and to correct the situation by preventing the horse from lifting out (10.8%). Auxiliary reins should also promote muscle development (8.8%, *n* = 32) and facilitate stance and flexion (6.6%, *n* = 24).

The participants were then asked whether they had noticed any defensive or feel-good reactions during the use of auxiliary reins in the last three months. In this regard, 92.8% (*n* = 335) stated that they had not noticed any signs of resistance. A minority (4.2%, *n* = 15) of the horses showed signs of tightening or bucking, or showed tense neck muscles and scuttling away (3%, *n* = 12). Tail flicking, lack of acceptance of the aids, stamping, and kicking were only occasionally reported. By contrast, many more feel-good signs were observed: snorting, swaying back (76.7%, *n* = 277), suppleness (56.8%, *n* = 261), swinging tail (41%, *n* = 148), and a satisfied facial expression (40.2%, *n* = 145) were mentioned in this context. Muzzle licking by their horses was noticed by 26.9% (*n* = 97) of the respondents.

Additionally, 84.4% (*n* = 350) of the test respondents stated that they did not have the impression that their horse had less air with auxiliary reins, and 97.5% (*n* = 353) of the horses did not make any breathing noise. On the other hand, seven participants felt that their horses breathed less, 1.4% (*n* = 5) noticed an increase in the breathing sound when using auxiliary reins, and 1.1% (*n* = 4) even noticed that it was created by the use of auxiliary reins.

**Figure 4:**
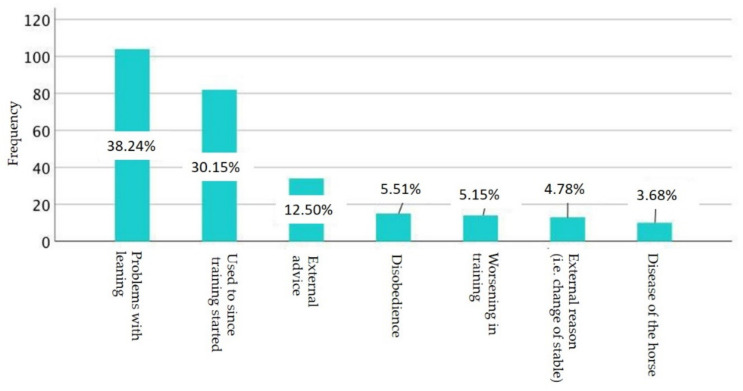
Frequency of reasons for the use of auxiliary reins.

A total of 55% (*n* = 199) of the respondents stated that they use auxiliary reins regularly. The reasons for this were that they had problems with leaning (52.33%, *n* = 104) or that they had been used to this since the training started (41.2%, *n* = 82). External advice also had an influence on the use of auxiliary reins for 17.1% (*n* = 13). A detailed description of further reasons is shown in Figure 4.

**Figure 5:**
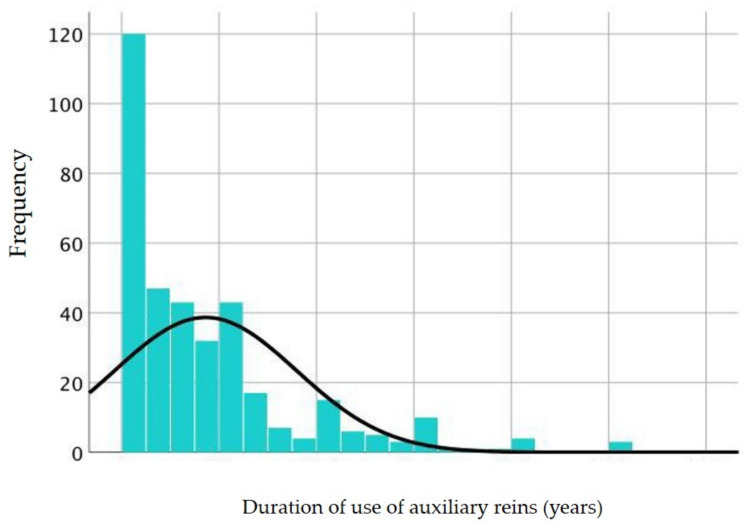
Frequency of duration of the use of auxiliary reins (in half-year steps as normal distribution).

Over half (52.8%, *n* = 105) of the respondents said that auxiliary reins would remain a permanent part of their equipment, while 27.6% (*n* = 55) wanted to use them until they felt they had become superfluous. When all respondents were asked how long they had been using auxiliary reins for their horse, they stated that they had been using them for an average of 4.3 ± 4.7 years (Figure 5).

**Figure 6:**
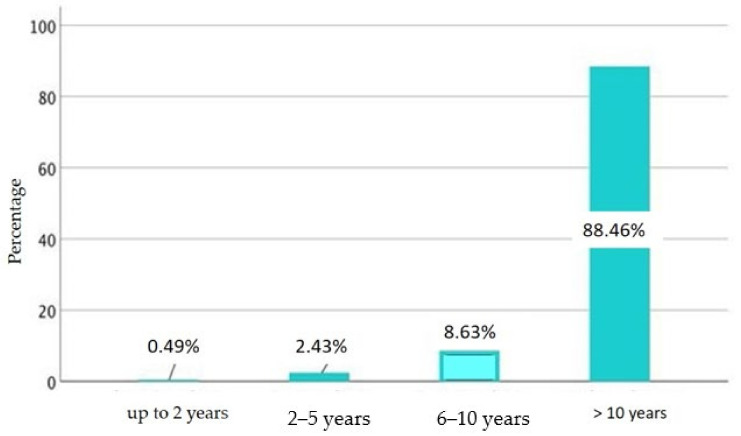
Experience of the respondents with horses in %.

Finally, personal details were requested. The respondents were 91.7% (*n* = 755) female and 1.8% (*n* = 15) male with an age range of 14 to 74 years (average 33.84 ± 11.32 years). The majority of participants were horse owners themselves (89.7%, *n* = 738). Out of all respondents, 22.5% (*n* = 185) worked with horses professionally; those who did not work with them took lessons from an independent trainer in 45.5% (*n* = 290) of the cases, 37.5% (*n* = 239) of which from an FN-licenced trainer. Of these, the participants received lessons once or twice a week (48.6%, *n* = 257) or less often (47.3%, *n* = 250). In some cases, lessons were given three to six times a week. Overall, 88.5% (*n* = 728) of the respondents had more than ten years of riding experience. Figure 6 provides a detailed overview.

The objective of using auxiliary reins was not significantly different between professional and recreational riders (chi-square test, *p* = 0.115, chi-square statistics 12.909). Both wanted to achieve contact, forward-downward extension, and support of the rider. However, by comparison, leisure riders wanted to avoid lifting the horse and to control the pace more often, whereas professional riders wanted to use auxiliary reins more to promote muscle development. The age of the horse and the reason for using auxiliary reins were also dependent on each other: starting training as a young horse was the reason for the use of auxiliary reins, especially among those under five years old (64.3%) and those five to nine years old (36.8%). With increasing age, however, leaning problems were mentioned more and more frequently; among the 15–19 year olds, they even accounted for 49.1%. The reasons disobedience (7.5%) and deterioration of training (5.7%) were also mainly provided regarding the 15–19-year-old horses.

Another significant association (Fisher’s exact test, *p* = 0.044, Fisher statistic 23.101) was found between the duration of the use of auxiliary reins and the intention to continue using them regularly. It can be stated that the riders who wanted to use auxiliary reins until the problem was solved or until the next training step did not use them for longer than four years. Even participants who did not use auxiliary reins depending on their feelings used them between zero and four years (80%). For most of the others, however, they remained an integral part of the equipment. Nevertheless, there was a significant association between the use of auxiliary reins and lessons with a trainer (chi-square test, *p* = 0.018, chi-square statistic 18.527). A little over half (50.1%) of the respondents who did not currently use auxiliary reins were taught by an independent or FN-licenced trainer. Only 2.5% of the latter used auxiliary reins daily and only 0.7% among independent trainers.

## 4. Discussion

*General aspects*—It is easy to reach many potential respondents of a target group by utilising online questionnaires. The good response rate of the present study confirmed this statement. It became repeatedly clear that some questions were not understood correctly; additionally, it must always be feared with predetermined answers that the participants will choose the answer that seems to them to be the right one and not the one that reflects their opinion.

Overall, the study population consisted predominantly of riders with significant experience with horses (almost 89% with more than 10 years). Moreover, almost 42% of the respondents participated regularly in competitions and 22.5% were even professionally involved with horses. This implies a great deal of experience. The majority of the participants took lessons with a trainer, which also imparts experience. Of course this is only a suggestion and it should be taken into account that the rider’s experience depends on the reasons for lessons and the level/experience of horse and rider.

*Use of the auxiliary reins—*Equine training aids are often used to influence the horse’s head and neck position and, by default, to impact spinal kinematics and stride length [1,6,13,14] or to increase the engagement of the horse’s core musculature and hind limbs to improve propulsion or generate muscle recruitment or hypertrophy [2,15]. The majority of the respondents in our study did not currently use auxiliary reins in training or did so less frequently than once or twice a week. This is certainly because about 72% of the respondents already had a trained horse and the use of auxiliary reins was not necessary. The FN [9] also advises the use of auxiliary reins in the course of training (when riding and on the lunge). However, some participants stated that they used auxiliary reins four to six times a week. This could be mainly the sliding ring martingale, which the interviewees said they generally used for safety reasons on the horse, or the side and running side reins if the horses were school horses. As auxiliary reins were considered important for many test riders, it is not surprising that 53% considered them to be fixed pieces of equipment. The participants predominantly used the type of reins corresponding to the different disciplines, especially sliding ring martingales for jumping and running side reins for lunging. In the present study, draw reins were the third most frequently used auxiliary reins for riding.

When horses were worked on the lunge line, 82% of the respondents used auxiliary reins for efficient training. The stable lateral guidance of the side reins and the flexibility of the lunging reins were underestimated. The FN [9] recommends these two auxiliary reins for the same reasons. When lunging, the running side reins were the ones most preferred by the interviewees, which is in line with the intention of achieving a forward-downward stretch in the horse. The fact that the horse is not able to find the necessary contact is underestimated [9]. Therefore, the lunge reins would be much more suitable for lunging as they can still provide sufficient contact. In our study, both the Chambon and Gogue reins were only intended for short-term use, if at all.

The sliding ring martingale is mainly used in show jumping. It enables the rider to keep control of the horse before the jump. The respondents always and continuously used it. The use of side, curb, running side, and draw reins for jumping, as occasionally stated by the participants in the study, must be clearly rejected. This is an abusive use because the horse no longer has the possibility of balancing itself over the jump. This is one of the reasons why this auxiliary rein is forbidden when jumping at competitions [3,4,5]. The study did not examine more closely what was required of the horses during training. In the classical school, auxiliary reins support work in hand [8]. However, draw reins, as indicated by the respondents, should not be used for this purpose. Similarly, the Gogue should also be rejected for this purpose.

*Handling the auxiliary reins during work—*The respondents mostly buckled the auxiliary reins after the warm-up phase. This allows the horse to move freely at first. Some even used the auxiliary reins later in the training, where they were only used for a very specific purpose. Some riders still buckled the reins on their horse in the stable aisle, which is a considerable safety risk for rider and horse and should be avoided [3,4,5]. In this context, it must also be critically evaluated that 50% of the survey participants did not adjust the auxiliary reins during training. This could be due to the fact that a sliding ring martingale was used, which only accounted for 23% of the auxiliary reins used. Therefore, ignorance or convenience must be assumed as the reason for this among the riders. The FN [9] recommends adjustment mainly after the warm-up phase. The setting of an inside position with adjustment after changing sides (as also carried out by some participants) should only be done with well-balanced horses, if at all [9]. It can be assumed that the participants wanted to achieve stance and flexion or counteract a pitching of the horse. However, this must be performed instead through specific balance exercises or accepting the lunge [9]. Considering time/length of training is also important.

Auxiliary reins were not loosened or removed after training by about one-fifth of the participants in the present study. By not removing or loosening the auxiliary reins, the trainer/rider is not allowing the horse to stretch/relax fully, which is not ideal during the warm down phase within exercise management of the horse.

In addition to the desire for leaning and forward-downward movement, some participants in the study also used auxiliary reins to achieve an arching of the back, which is only secondary to the use of auxiliary reins. It is preceded by leaning and suppleness, which can be promoted by the use of auxiliary reins. Lunging with auxiliary reins can also provide a certain amount of strength training, but the auxiliary reins themselves do not help build muscle [16]. They provide support so that the horse can swing from the back to the front and push against the bit.

*Reasons for using auxiliary reins—*Generally, horse training can be divided into two categories: positive or negative reinforcement. For learning to be effective, the rider or handler must be able to give clear signals and respond quickly, within seconds, when the horse offers the ‘correct’ response or behaviour, for the horse to link the stimulus to the desired response. Therefore, to use training aids responsibly, the handler or rider must consider the mechanism of action of the equipment, how skilled they are in working the horse within the aid, and how the horse will interpret and learn from the use of the equipment. Depending on this, the use of training aids can be beneficial, but their incorrect use or misuse can be very detrimental to equine training and welfare and result in conflict behaviours.

The use of auxiliary reins as an equine training aid may have subsequent benefits, such as placing the horse in a way of going that promotes muscle development, enhances symmetry, or improves the horse’s quality of work [1,2,3,4,5,6,12]. Though the persistent use of draw reins will likely result in a lowered head and neck position through a pulley action, this will not teach the horse to work in self-carriage if there is no removal of pressure to teach the horse the desired response to the rein aid [17,18,19,20]. A remaining problem of auxiliary reins is mainly that they can be buckled too tightly, forcing the horse behind the vertical, what is called “hyperflexion”, and results in various negative effects. Thus, the key points for using auxiliary reins correctly are the riders’s/user’s skills and experiences.

In our study, the assumption that the respondents had sufficient experience in the use of auxiliary reins (experience over 10 years) contrasts with the result that more than half of the participants in our study used them for their own support. Further investigations addressing this point are necessary.

Most of the participants use a sliding ring martingale to prevent the horse from raising its head too high and pulling away. Other auxiliary reins are also used to counteract this. If, however, the horse moves against the reins and tightens its back, the result will be a tensing of the lower neck muscles with a loss of contact. The majority of the study participants wanted to achieve better flexion and, thus, were aware of the benefits of auxiliary reins.

A small number of the participants admitted to having problems with the horse when using auxiliary reins. Whether this could be attributed to incorrect use or other circumstances cannot be determined. Auxiliary reins can also generally correct faulty behaviour in the horse and do not necessarily have to be responsible for it [9]. Even if only a few participants had the impression that their horse was breathing less easily when using auxiliary reins, this should always lead to changing the training or seeking professional advice elsewhere. An excessively tight buckling of auxiliary reins could lead to the development of breathing noises or to their amplification [21]. The survey showed that over half of the respondents used auxiliary reins at least once a week. Auxiliary reins can be used at the beginning of work to counteract problems with rideability, leaning difficulties, mounting, bucking, and lack of suppleness [9]. Many participants in the study also provided such reasons for the regular use of auxiliary reins. About 17% followed an external recommendation. The extent to which this may have been professional advice cannot be determined at this point. The FN recommends the use of auxiliary reins in the training of young horses, especially for lunging, in order to familiarise them with the subsequent contact with the rider’s hand [3,4,5]. However, since the average age of a horse is 12 years, it seems reasonable to assume that many participants have always used auxiliary reins and not moved away from them. This could also be reflected in the fact that about 58% of participants wanted to continue using auxiliary reins.

Professional and leisure riders were first compared in the investigation of statistically proven associations. Both groups pursued the same goals with the use of auxiliary reins (contact, forward downward stretching, support for the rider). In the case of leisure riders, however, there was more often the desire to prevent the horse from being lifted out of the saddle and to be able to control the pace or correct misbehaviour better. This suggests that professional riders were particularly aware of the sometimes negative consequences of the permanent use of auxiliary reins and that no success can be achieved in the long term as a result [3,4,5]. On the other hand, professional riders stated that they wanted to specifically promote muscle development with the use of auxiliary reins, which cannot be achieved with the reins alone but requires good training [16]. The training of young horses usually begins at the age of three. In the present work, this was the predominant reason why auxiliary reins were used in this age group. Many horses were also trained at the age of five to nine years, which explains the use in this group. With increasing age, however, leaning problems, disobedience, and deterioration in training were increasingly provided as reasons. However, leaning problems were also provided as a reason for the younger horses, which is surprising as this is more of a problem for older horses. Thus, the goal set by the FN of making the auxiliary reins superfluous as training progresses was once again not achieved: auxiliary reins remained an integral part of the equipment for 44% of the participants [3,4,5]. Even among the participants who had been using auxiliary reins for more than five years, the majority did not want to do without them. The survey participants who took lessons with an independent trainer did so without auxiliary reins more often than those who received lessons from an FN-licenced trainer. Whether this reflects a seat training on the lunge by the trainer can only be assumed, but it would explain the situation. This would then also be in the spirit of the FN recommendations [9]. If the sliding ring martingale is used more than three times a week, its use must be suspect. Independent trainers, on the other hand, often claimed to train horses freely and without coercion. However, it is not always clear whether sufficient expertise was available as certification is lacking [22]. Similarly, the use of auxiliary reins was not always to be equated with coercion. Only in a few cases did the participants say that they tied out their horse severely behind the vertical and, thus, noticed that the air supply was restricted, which is what studies have stated [21].

The majority of the participants in the study felt they had sufficient knowledge about the use of auxiliary reins attributed to their experience (years ridden) as a rider. Even though many did not want to do without the sliding ring martingale in the long run, there was only a slight risk for the horse and the general recommendations of the FN were only marginally contradicted. More serious violations, on the other hand, were only recognisable in a small section of the respondents. However, against the background of animal welfare, these are to be taken very seriously in some cases and should, therefore, be stopped immediately. How this can best be achieved could be the subject of further research. The goal must always be to use auxiliary reins only for a short time in a problem-oriented way in the training and correction of horses in order to make these reins superfluous, and this knowledge must become an integral part of riding training. Further examinations should focus on the distinction of a running (sliding ring) martingale versus the other auxiliary aids examined, as many riders would use the former as a standard piece of tack throughout a horse’s career.

*Consequences of the invalid use of auxiliary reins*—Equitation science focuses on a thorough understanding of both equine ethology and learning theory. This combination leads to more effective horse training and plays a role in keeping horse riders and trainers safe around horses [23]. Scientific research and evidence of the impact of auxiliary reins as training aids on performance, gait characteristics, proprioception, the horse’s biomechanics, as well as the equine behaviour, positive/negative learning experience, and welfare is limited [12,21,24,25,26,27]. If, when using side reins (even when fitted and used correctly), the pressure of the rein is not removed if the horse gives the desired response, the reins can encourage hyperflexion as the horse seeks a release from a consistent contact.

Improper use with auxiliary reins buckled too tightly in order to force the horse behind the vertical (hyperflexion) can result in negative biomechanical and health-influencing aspects. Hyperflexion tightens the nuchal ligament, shifts the horse’s weight to the hindquarters, and is accompanied by a reduction of the fetlock joint angle, which could result in lameness [1,13,14,28,29,30,31]. When horses that are still untrained work against the forced posture, pain can occur in the long back muscle with a faulty (crooked, stiff) tail posture [22]. The larynx could also be severely affected by hyperflexion [32,33]. Unwelcome problems with horses can develop because of inappropriate application of training techniques and/or training aids. The welfare implications of behavioural problems in general suggest that horse handlers and riders should become conversant in learning theory since it is the basis of good training [17]. Traditional methods currently used in training horses are predominantly based on negative reinforcement, causing much resistance and conflict for the horse. However, not all negative reinforcement results in conflict and resistance. Learning theory provides the clearest basis for good animal training. When using equipment such as auxiliary reins, the use of negative reinforcement cannot be avoided [23].

There are behavioural consequences of physical restraint in horses through using auxiliary reins. It is clear that sliding ring martingales and tie-downs that apply pressure to the nasal planum via the noseband (in the case of the standing sliding ring martingale and tie-downs) or the mouth via the reins (in the case of the running sliding ring martingale or draw reins) are designed to prevent evasive raising of the head [18,19]. The rider can use the lever action of the running sliding ring martingale to pull the head lower. Critics rightly point out that these reins force the horse into an outline rather than train self-carriage through lightness. When the head is forced downwards, the muscles of the neck and top line are not ‘suspending’ the head and neck, but instead, the horse is attempting to raise its head against aversive pressure [20,34].

*Limitations*—There is a possibility that especially those who are critical of the use of auxiliary reins would take part in such a questionnaire campaign. This should be taken into account when evaluating the results. Additionally, further research should go more in detail and the research should focus more on individual auxiliary reins and consider positive as well as negative effects and proposed function both for the rider/user and for the training of the horse. Lastly, increased emphasis should be placed on the well-being of the horses (e.g., use of the Horse Grimme scale or other pain scores).

## 5. Conclusions

Auxiliary reins are often criticised if they are used incorrectly, which can harm the horse by causing overwork, accidents, and injuries. Most participants of our study felt they used the reins responsibly. However, to our mind, there is still a need for clarification and a lack of information among the horse owners regarding the pros and cons of using auxiliary aids as well as the effects of the different reins used. Half of the participants did not change the reins during the entire training session. Some participants adjusted their horse too tightly and did not change anything despite the related breathing problems. Further research is needed to explore strategies to educate horse owners/trainers, improve their knowledge about riding aids and, by association, improve equine wellbeing. Educating horse owners, riders, and trainers by promoting scientific information and research results is essential to promote equine wellbeing.

## Data Availability

Not applicable.
